# Fellow-Workers and the Roll of Honour

**Published:** 1916-01-01

**Authors:** 


### ROUND THE WORLD.

[The Editor invites Early Information and Contributions Marked "Fellow-Workers."]

### Fellow-Workers and the Roll of Honour.

COLONEL ERNEST OCTAVIUS WIGHT, who, it is officially reported, was killed in action in Flanders on December 19, was Assistant Director of Medical Services, 49th Division. He entered the Service in 1882 and reached the rank of lieutenant-colonel in the R.A.M.C. in 1902, retiring in 1907. On the outbreak of war he offered himself for re-employment, and was gazetted Assistant Director of Medical Services in April of this year. Colonel Wight saw service at Lushai (1892) and was decorated with the medal with clasp.

CAPTAIN H. J. BURKE, L.R.C.P.I., L.R.C.S.I., R.A.M.C., attached 1st/8th Battalion West Yorkshire Regiment (T.F.), has had conferred upon him the Military Cross for conspicuous gallantry. A sergeant in the front line had his leg crushed by the blowing in of a dug-out, and Captain Burke found immediate amputation necessary. In order to save time he crawled across the open to get his instruments, while the enemy turned a machine-gun on him. In spite of their fire he returned the same way and coolly performed the operation in the trench while the enemy were shelling it heavily.

CAPTAIN B. J. HACKETT, M.B., R.A.M.C., attached 7th Battalion Suffolk Regiment, who has been awarded the Military Cross for conspicuous gallantry and devotion to duty at Loos, was formerly resident medical officer at H.M. Prison, Mountjoy, Dublin, and had held a resident appointment at St. Vincent's Hospital, Dublin. It is recorded that when the battalion to which he was attached had suffered very heavy casualties and had run out of dressings, Captain Hackett brought up a fresh supply from the dressing station, crossing over about 1,000 yards in the open. He has frequently attended the wounded under fire, and has shown great bravery.

LIEUTENANT J. R. G. GARBUTT, M.B., R.A.M.C., attached 8th Battalion, K.O. Scottish Borderers, who has been killed in action, was gazetted temporary lieutenant twelve months ago. Before the war he held a resident appointment at the General Infirmary, Macclesfield. He received the M.B. degree at Aberdeen University in 1911.

DR. F. M. SANDWITH, who has been appointed consulting physician to His Majesty's troops in the Mediterranean and is leaving shortly for Egypt, is Gresham Professor of Physic and senior physician to the London School of Tropical Medicine. Dr. Sandwith served as ambulance surgeon in the Turco-Serbian War 1875, and in the Russo-Turkish campaign 1877-1878. He was present at the fighting at Shipka Pass, and served on Baker Pasha's staff during his retreat across the Rhodope Mountains. He proceeded to Egypt for the cholera epidemic in 1883, and became Vice-Director of the Sanitary Department of the Egyptian Government until 1885 and Professor of Medicine at the Egyptian Government School of Medicine till 1903. During the South African War he was senior physician of the Imperial Yeomanry Hospital at Pretoria.

LIEUTENANT-COLONEL A. W. SHEEN, R.A.M.C. (T.F.), who with his principal assistants will form the nucleus of the staff of the new Welsh War Hospital, has been an officer in the Army for twenty years, and was mentioned in dispatches in the South African War. In private practice he is well known as a consulting surgeon.

LIEUTENANT J. M. M. MARSHALL, who was killed on active service, is the subject of a note in the December issue of the *St. Bartholomew's Hospital Journal.* While repairing a part of the parapet he was shot by an enemy sniper; the bullet entered his left arm and passed through his side, penetrating his lung.

LIEUTENANT A. HEGARTY, R.A.M.C., who was killed in action in France on December 16, was the fourth son of Mr. C. Hegarty, of Magherafelt, Co. Londonderry. Lieutenant Hegarty obtained his medical qualification at Dublin University this year, and received a commission in July last, being attached to a battery of Royal Field Artillery.

LIEUTENANT-COLONEL L. F. CHILDE, whose death has occurred at Southsea, obtained a commission in the Indian Medical Service in 1887, and was on military duty till 1889, in which year he was appointed to a position at the Sir J. Jejeebhoy Hospital, Bombay, becoming in due course senior medical officer. He was also Principal and Professor of Medicine at the Grant Medical College, Bombay, and at one time Dean of the Medical Faculty at the University of Bombay. He took his M.B. degree at London University in 1885, and for some time held a resident appointment at Guy's Hospital.

COLONEL RODERICK MACRAE, whose death was recently announced in the *Morning Post,* was a former Inspector-General of Civil Hospitals in Bengal. He graduated M.B., C.M. at Edinburgh University in 1873. Obtaining a commission in 1875, he was in military employ till April 1882, and during that period served in the Afghan War, receiving the medal with clasp. Afterwards he held civil surgeoncies at various places in Bengal, reached the rank of colonel in February 1905, and in March was appointed Inspector-General of Civil Hospitals, and Sanitary Commissioner for Burma in 1906, being promoted to be Inspector-General of Civil Hospitals, Bengal, and holding that position till his retirement in 1910.

LIEUTENANT H. M. SCOTT, R.A.M.C., attached 11th Lancashire Fusiliers, who has been wounded, received his medical training at the London Hospital, and obtained his joint diploma M.R.C.S., L.R.C.P. in 1905. He has held resident appointments at the London Hospital.

IN memory of the late Captain Bingham, M.R.C.S., L.R.C.P., his medical colleagues have affixed a fine memorial to the outer wall of the Royal Lancaster Infirmary. He was captain in the 5th Battalion (T.F.) of the Royal Lancaster Regiment. He went out with the battalion to the Front, and was one of the few officers who escaped without injury in the operations which covered the battalion with renown. He was invited to join the R.A.M.C., but preferred to remain with his battalion as a combatant. He was home on short leave in May, and in the early morning of the day following his arrival back at the Front he was shot by a German sniper as he and other officers were leaving some new trenches they had been reconnoitring. For ten or eleven years Dr. Bingham had been in Lancaster and the neighbourhood.

